# Novel multi-targeted ErbB family inhibitor afatinib blocks EGF-induced signaling and induces apoptosis in neuroblastoma

**DOI:** 10.18632/oncotarget.13657

**Published:** 2016-11-26

**Authors:** Xinfang Mao, Zhenghu Chen, Yanling Zhao, Yang Yu, Shan Guan, Sarah E. Woodfield, Sanjeev A. Vasudevan, Ling Tao, Jonathan C. Pang, Jiaxiong Lu, Huiyuan Zhang, Fuchun Zhang, Jianhua Yang

**Affiliations:** ^1^ Xinjiang Key Laboratory of Biological Resources and Genetic Engineering, College of Life Science and Technology, Xinjiang University, Urumqi 830046, P. R. China; ^2^ Texas Children's Cancer Center, Department of Pediatrics, Dan L. Duncan Cancer Center, Baylor College of Medicine, Houston, Texas 77030, USA; ^3^ Department of Ophthalmology, Shanghai Tenth People's Hospital, Tongji University School of Medicine, Shanghai 200072, P. R. China; ^4^ Division of Pediatric Surgery, Texas Children's Hospital Department of Surgery, Michael E. DeBakey Department of Surgery, Dan L. Duncan Cancer Center, Baylor College of Medicine, Houston, Texas 77030, USA

**Keywords:** neuroblastoma, EGFR inhibitor, afatinib, apoptosis, chemotherapy

## Abstract

Neuroblastoma is the most common extracranial solid tumor in children. The ErbB family of proteins is a group of receptor tyrosine kinases that promote the progression of various malignant cancers including neuroblastoma. Thus, targeting them with small molecule inhibitors is a promising strategy for neuroblastoma therapy. In this study, we investigated the anti-tumor effect of afatinib, an irreversible inhibitor of members of the ErbB family, on neuroblastoma. We found that afatinib suppressed the proliferation and colony formation ability of neuroblastoma cell lines in a dose-dependent manner. Afatinib also induced apoptosis and blocked EGF-induced activation of PI3K/AKT/mTOR signaling in all neuroblastoma cell lines tested. In addition, afatinib enhanced doxorubicin-induced cytotoxicity in neuroblastoma cells, including the chemoresistant LA-N-6 cell line. Finally, afatinib exhibited antitumor efficacy *in vivo* by inducing apoptosis in an orthotopic xenograft neuroblastoma mouse model. Taken together, these results show that afatinib inhibits neuroblastoma growth both *in vitro* and *in vivo* by suppressing EGFR-mediated PI3K/AKT/mTOR signaling. Our study supports the idea that EGFR is a potential therapeutic target in neuroblastoma. And targeting ErbB family protein kinases with small molecule inhibitors like afatinib alone or in combination with doxorubicin is a viable option for treating neuroblastoma.

## INTRODUCTION

Neuroblastoma (NB) is a pediatric cancer deriving from neural crest and is commonly found in the adrenal medulla or along the sympathetic chain [1]. As the most common extracranial solid pediatric tumor, NB causes approximately 13% of mortality from all pediatric malignancies [2, 3]. NB are stratified into five risk groups, 1, 2, 3, 4, and 4S, and late stages with *MYCN* amplification have been defined as “high-risk” [4]. Despite the improvements in treatment made in recent decades, the cure rate for high-risk NB patients remains disappointingly low with a five-year survival rate less than 50% [5, 6]. The poor outcomes warrant investigation for a better biological understanding of this pediatric malignancy and development of new therapeutic targets and treatment options to cure this disease.

The ErbB family of RTKs, which consist of Epidermal growth factor receptor (EGFR) (ErbB1 or HER1), ErbB2 (HER2 or Neu), ErbB3 (HER3), and ErbB4 (HER4), have been shown to promote tumor progression in various cancer types [7]. Of the human ErbB family members, EGFR functions as a critical mediator of tumor progression in several cancer types. Notably, abnormally activated EGFR predicts poor outcomes in many cancer types including non-small-cell lung cancer (NSCLC), head and neck cancer, and breast cancer [8–11]. In addition, somatic mutations of *EGFR* lead to continuous activation of kinase activity, resulting in uncontrolled cell division and tumorigenesis [12–14]. EGFR is a transmembrane tyrosine kinase receptor that binds to ligands like Epidermal growth factor (EGF) and Transforming growth factor alpha (TGF-α) through its extracellular domain to activate downstream signaling pathways [15, 16]. These pathways include PI3K/AKT/mTOR pathway, which is critical for cell survival and proliferation, as well as, the KRAS/BRAF/MEK/ERK, and JAK2/STAT3 pathways [7, 17, 18]. Moreover, EGFR has been found to be widely expressed in NB cells and primary tumors, and activation of EGFR significantly promoted NB cell proliferation [19, 20]. Previous studies have identified EGFR as a potential therapeutic target in NB [21] and pan-ErbB inhibition is a therapeutic option for treating NB patients [20], which supports further study of the efficacy of ErbB family inhibitors in NB.

The pan-ErbB family tyrosine kinase inhibitor afatinib (BIBW-2992, trade name Gilotrif, previously Tomtovok and Tovok) has been approved by the U.S. Food and Drug Administration (FDA) for first-line treatment of patients with NSCLC with distinct EGFR mutations [22]. In cell-free assays, afatinib shows potent activity against the proteins encoded by wild-type and mutant *EGFR* and *HER2* including the L858R and T790M *EGFR* mutations [23]. In addition, afatinib shows inhibitory effects on cells with wild-type *HER4* [24, 25]. Afatinib exhibits potent antitumor effects against various types of carcinomas including breast cancer, head and neck squamous cell cancer, colorectal cancer and NSCLC [26–28]. In addition, EGFR and HER4 are known to be expressed in NB cell lines and patient samples and HER2 in NB patient samples. Thus, investigation of the efficacy of afatinib in NB is warranted [20]. However, to our knowledge, the antitumor effects of afatinib on NB have not yet been explored.

In this study, we investigated the anti-tumor effects of ErbB family member inhibitor afatinib on NB. We found that afatinib inhibited the cell viability and induced apoptosis in NB cells. In addition, afatinib blocked EGF-induced activation of PI3K/AKT/mTOR signaling in all NB cell lines tested. Moreover, afatinib sensitized a subset of NB cells to doxorubicin treatment. More importantly, afatinib induced apoptosis and blocked PI3K/AKT/mTOR signaling in an orthotopic xenograft NB mouse model. Taken together, our study supports the idea that EGFR is a potential therapeutic target in NB and treating NB patients by ErbB family protein kinases inhibitors like afatinib alone or in combination with doxorubicin is a promising strategy.

## RESULTS

### The prognostic significance of EGFR expression in NB

**Figure 1:**
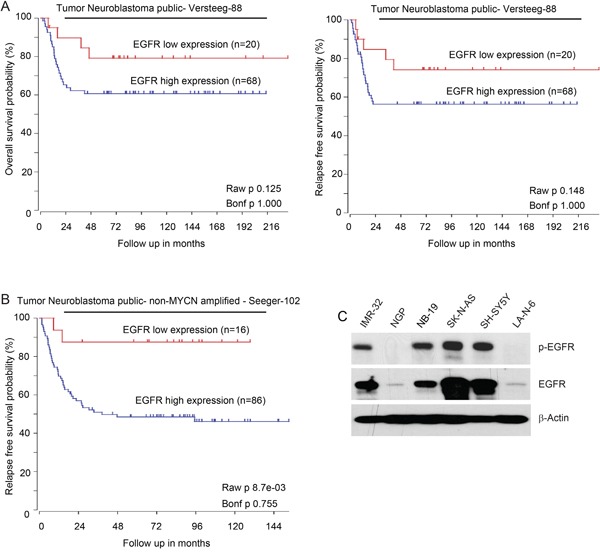
High expression of EGFR predicts poor outcomes in NB patients **A.** Overall survival probability and Relapse-free survival probability for NB patients with high EGFR expression (blue; n=68) and low EGFR expression (red; n=20) (Versteeg-88 data set). **B.** Estimated relapse-free survival rates in NB patients with high EGFR expression (blue; n=86) and low EGFR expression (red; n=16) from Seeger-102 data set. **C.** Basal expressions of phospho-EGFR (Y1068) and total EGFR in a panel of six NB cell lines: IMR-32, NGP, NB-19, SK-N-AS, SH-SY5Y and LA-N-6. The expression pattern of p-EGFR and EGFR varies in those cell lines.

Aberrant activated expression of EGFR correlates with poor outcomes in many adult malignancies [8–10]. Hence, we first evaluated the clinical significance of *EGFR* expression in NB patients. Data analysis of the R2 database (R2: http://r2.amc.nl) reveals that high expression of *EGFR* predicts lower overall and relapse-free survival in the Versteeg-88 data set (Figure 1A). In addition, consistently, high expression of *EGFR* is associated with lower relapse-free survival in *MYCN* non-amplified NB patients from the Seeger-102 data set (Figure 1B). These data suggest that EGFR is a potential biomarker for the prediction of outcomes in NB patients. We then examined the endogenous expression level of EGFR in a subset of NB cell lines. A protein immunoblotting assay revealed the expression pattern of EGFR in the six NB cell lines (IMR-32, NGP, NB-19, SK-N-AS, SH-SY5Y, LA-N-6) tested. As shown in Figure 1C, high expression levels of phospho-EGFR (Y1068) and total EGFR were detected in SK-N-AS, SH-SY5Y, IMR-32, and NB-19 cells, whereas NGP and LA-N-6 cell lines showed very low expression of phospho-EGFR (Y1068) and total EGFR.

### Afatinib shows cytotoxic effect on NB cells

**Figure 2:**
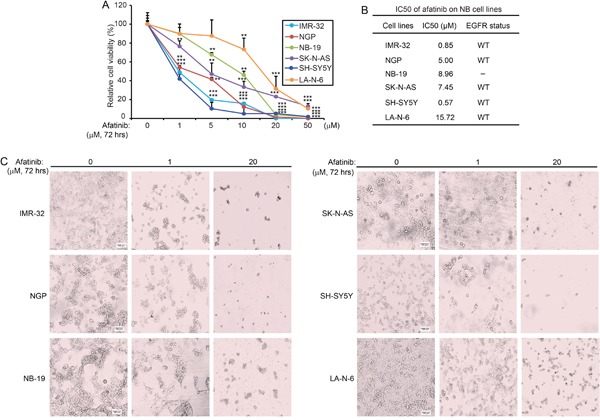
Afatinib shows cytotoxic effect on NB cells **A.** Six NB cell lines (IMR-32, NGP, NB-19, SK-N-AS, SH-SY5Y and LA-N-6) were treated with increasing concentrations of afatinib for 72 hrs. Cell viability was then assessed by a CCK-8 assay. Data were presented as mean ± SD. *P* <0.01 (**), or *P* <0.001 (***) (Student's t-test) were indicated. **B.** The IC50 values of afatinib on the tested NB cell lines and *EGFR* status of each cell line were listed. Five of the six NB cell lines were *EGFR* wild-type (WT). **C.** Morphologic changes of the six NB cell lines treated with afatinib for 72 hrs were shown.

To assess the cytotoxicity of afatinib on NB cell lines, six NB cell lines (IMR-32, NGP, NB-19, SK-N-AS, SH-SY5Y, LA-N-6) were treated with increasing concentrations of afatinib for 72 hrs. Afatinib significantly inhibited the cell viability of the NB cell lines tested in a dose-dependent manner (Figure 2A). The IC50 values of afatinib on NB cell lines and *EGFR* status of those cell lines were listed (Figure 2B). We found that IMR-32 and SH-SY5Y cells were very sensitive to afatinib with an IC50 of 0.85 μM and 0.57 μM, respectively. LA-N-6 cells were the most resistant to afatinib treatment, with an IC50 of 15.72 μM. This suggests that afatinib has high efficacy against NB cell lines. Morphological changes of the treated cells further confirmed the cytotoxicity of afatinib on the NB cells (Figure 2C). These data demonstrate that afatinib can inhibit the cell viability of NB cell lines in a dose dependent manner.

### Afatinib suppresses the anchorage-independent growth of NB cells

**Figure 3:**
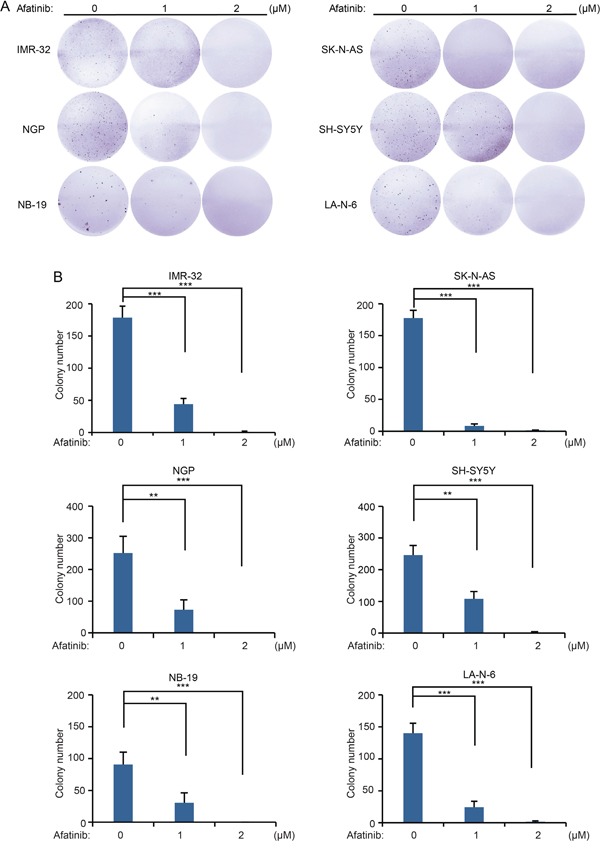
Afatinib suppresses the anchorage-independent growth of NB cells **A.** A panel of six NB cell lines were seeded in six-well plates with indicated concentrations of afatinib in soft agar, and grown for 2 to 3 weeks, followed by staining with crystal violet for 4 hrs and the photos were taken. **B.** Colonies were counted and colony numbers were presented as mean ± SD. *P* <0.01 (**), or *P* <0.001 (***) (Student's t-test) were indicated.

To evaluate whether afatinib affects the anchorage-independent growth ability of NB cells, a soft agar assay was performed in which cell growth independent of a solid surface is measured. Lower doses of the inhibitor were used in this assay to measure effects on growth in the absence of cytotoxicity. As expected, afatinib treatment led to decreased colony formation ability of all the tested NB cell lines in a dose dependent manner (Figure 3A). Quantification of the results from this assay show that afatinib significantly suppressed the anchorage-independent growth of NB cells (Figure 3B).

### Afatinib induces apoptosis in NB cells

**Figure 4:**
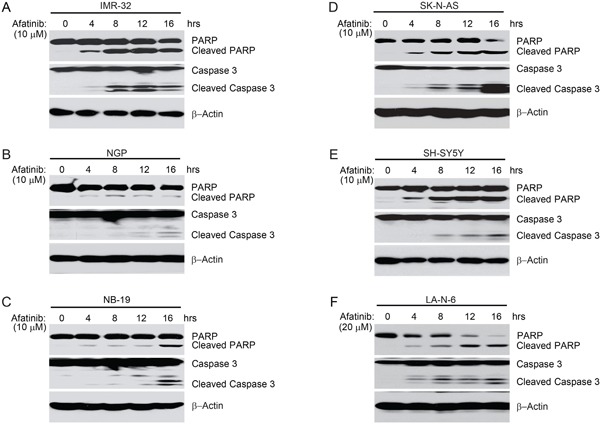
Afatinib induces apoptosis in NB cells **A-F.** IMR-32, NGP, NB-19, SK-N-AS and SH-SY5Y and LA-N-6 cells were treated with afatinib (10 μM or 20 μM) for various time points (0-16 hrs). At the end of treatment, cells were harvested and cell lysates were subjected to SDS-PAGE, and then immunoblotted with the indicated antibodies. β-Actin was used as a loading control.

Previous studies reported that afatinib can inhibit cell proliferation and induce apoptosis in various types of cancer cells [29–31]. To explore whether afatinib could induce apoptosis in NB cells, IMR-32, NGP, NB-19, SK-N-AS, SH-SY5Y, and LA-N-6 cells were treated with afatinib for various time points (0-16 hrs). We found that afatinib induced apoptosis in all the tested NB cell lines in a time-dependent manner, as shown by PARP and Caspase 3 cleavage (Figures 4A-4F).

### Afatinib effectively inhibits the EGF-induced PI3K/AKT/mTOR signaling pathway

**Figure 5:**
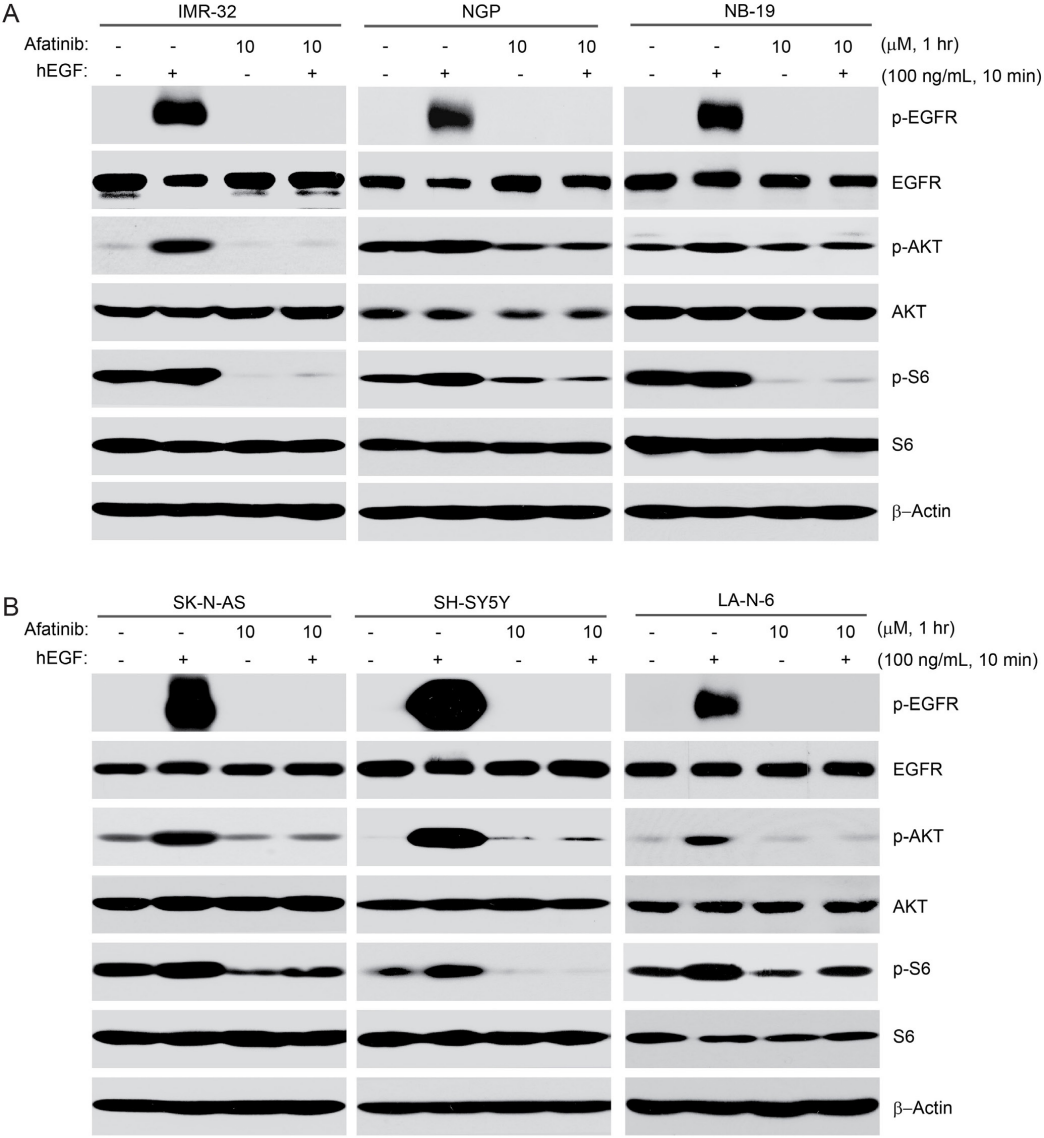
Afatinib blocks EGF-induced phosphorylation of EGFR, AKT and S6 in NB cells **A-B.** Six NB cell lines (IMR-32, NGP, NB-19, SK-N-AS, SH-SY5Y, and LA-N-6) were starved for 16 hrs in serum-free medium before exposed to afatinib (10 μM) treatment for 1 hr. Then the cells were stimulated with or without 100 ng/ml hEGF for 10 min. Cells were then collected and subjected to SDS-PAGE, immunoblotted with the indicated antibodies, respectively. β-Actin was used as a loading control in all samples.

Human EGF (hEGF) has been reported to bind to and activate EGFR, which leads to activation of the PI3K/AKT/mTOR signaling pathway [32]. The PI3K/AKT/mTOR pathway promotes cell growth and proliferation in many tumor types and is the most important downstream signaling pathway mediated by EGFR in NB cells [7, 21]. As afatinib potently inhibits the activity of both wild-type and mutant (L858R/T790M) EGFR [23], we hypothesized that the inhibitor may block EGF-induced activation of EGFR and its downstream signaling. To test this hypothesis, six NB cell lines (IMR-32, NGP, NB-19, SK-N-AS, SH-SY5Y, LA-N-6) were used in an EGF stimulation assay in which they were starved in serum-free medium for 16 hrs and then exposed to 10 μM afatinib with or without hEGF treatment. As predicted, afatinib dramatically blocked hEGF-induced phosphorylation of p-EGFR (Y1068), p-AKT (S473) and p-S6 (S235/236) in all the NB cell lines tested (Figures 5A-5B). These results suggest that afatinib effectively inhibits EGF-induced activation of EGFR and its downstream PI3K/AKT/mTOR signaling pathway in NB cells.

### Afatinib enhances doxorubicin-induced cytotoxicity in NB cells

**Figure 6:**
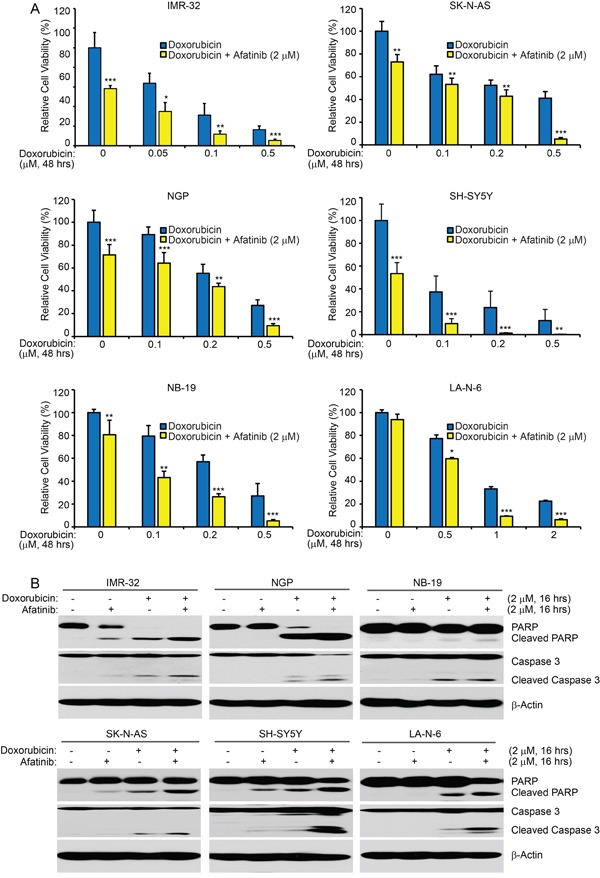
Afatinib enhances doxorubicin-induced cytotoxicity in NB cells **A.** Six cell lines were seeded in 96-well plates and were incubated with doxorubicin at the indicated concentrations with or without afatinib (2 μM) for 48 hrs. Cell viability was then measured by CCK-8 assay. Data were represented as mean ± SD. *P* <0.05 (*), *P* <0.01 (**) or *P* <0.001 (***) (Student's t-test) are indicated. **B.** IMR-32, NGP, NB-19, SK-N-AS, SH-SY5Y, and LA-N-6 cells were treated with either doxorubicin (2 μM) alone, afatinib (2 μM) alone, or their combinations for 16 hrs. Then whole cell lysates were then subjected to SDS-PAGE and immunoblotted with the PARP and Caspase 3 antibodies. β-Actin was used as a loading control in all samples.

Since afatinib was able to inhibit cell proliferation and induce apoptosis in NB cells, we reasoned that the combination therapy of afatinib and the traditional therapeutic agent doxorubicin may increase the chemo-sensitivity of NB cells to doxorubicin treatment. We found that afatinib (2 μM) sensitized all six NB cell lines tested to doxorubicin treatment, compared with the single drug treatment of doxorubicin (Figure 6A). Moreover, afatinib (2 μM) enhanced doxorubicin (2 μM)-induced apoptosis, as shown by increased levels of PARP and Caspase 3 cleavage (Figure 6B). Importantly, afatinib significantly enhanced both doxorubicin-induced apoptosis and inhibition of cell proliferation in the chemoresistant LA-N-6 cells (Figures 6A-6B). Taken together, these data demonstrate that afatinib enhances doxorubicin-induced cytotoxicity in NB cells.

### Afatinib induces apoptosis and blocks the activity of PI3K/AKT/mTOR signaling in an orthotopic xenograft NB mouse model

**Figure 7:**
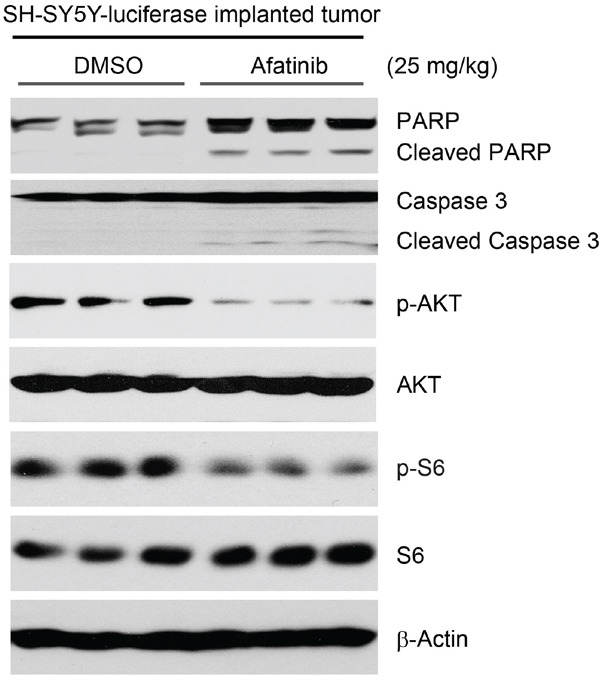
Afatinib induces apoptosis by blocking PI3K/AKT/mTOR signaling in an orthotopic xenograft NB mouse model The mice bearing SH-SY5Y-luciferase cells xenografted tumors for four weeks were treated with either afatinib (25 mg/kg) or an equal volume of DMSO by i.p. injection daily for three days. Four hours after the last treatment, the mice were sacrificed and the tumors were harvested and lysed for immunoblotting with the indicated antibodies. β-Actin was used as a loading control.

To explore the antitumor effects of afatinib *in vivo*, an orthotopic xenograft NB mouse model was used. Mice bearing SH-SY5Y-luciferase xenografted tumors were treated with either afatinib (25 mg/kg) or an equal volume of dimethyl sulfoxide (DMSO) daily for three days by intraperitoneal (i.p.) injection. At the end of treatment, the mice were sacrificed and the tumors were harvested and lysed for a protein immunoblotting assay. As shown in Figure 7, afatinib induced apoptosis in NB tumor cells, as shown by cleavage of PARP and Caspase 3. Furthermore, the phosphorylation levels of AKT and S6 were much lower in the afatinib treated group, compared with the DMSO control group (Figure 7). Together, these data indicate that afatinib induces apoptosis and blocks PI3K/AKT/mTOR signaling activity *in vivo*.

## DISCUSSION

Aberrant EGFR activation has been shown to be associated with the tumorigenesis of a variety of malignancies, including NB [33, 34]. EGFR is present in many tumors from NB patients and is rarely mutated [35–37]. Recently, a novel *EGFR* extracellular domain deletion mutant *EGFR*^Δ768^ has been found in primary tumors of NB patients and in a NB cell line BE2M17, which confers an aggressive cancer phenotype in NB cells [38]. Another known *EGFR* mutant *EGFRvIII* (*EGFR*^Δ801^) has also been found in NB patients [38]. While both of the two *EGFR* mutants are constitutively active and able to activate downstream signaling cascades, the biologic and biochemical properties are distinctly different. Besides, mutation analysis of 106 NB patients revealed that no mutations in the *EGFR* gene in the examined group of NB patients, and only three polymorphisms were identified in the *EGFR* gene (c.2184+19 G>A, c.2361 G>A and c.2508 C>T) [35]. There were no associations between EGFR expression and gene polymorphisms either. In addition, *EGFR* is amplified in the SK-N-AS cell line used in this study, according to The Cancer Genome Atlas (TCGA) data portal (http://www.cbioportal.org/). This is consistent with the expression level of EGFR in SK-N-AS cells, as shown in Figure 1C. In this study, we found that high expression of EGFR predicts poor outcome in two datasets of NB patients. Moreover, IMR-32, NB-19, and SH-SY5Y cell lines also showed relatively high expression of EGFR, whereas the endogenous levels of EGFR in NGP and LA-N-6 cell lines were very low. Taken together, our data support that EGFR is a potential therapeutic target in NB.

The novel multi-target small molecule inhibitor afatinib was effective on HER2 and several forms of EGFR in a cell-free assay [23]. Consistent with previous studies, we found that EGFR was expressed in all the NB cell lines tested [20]. In addition, the PI3K/AKT/mTOR pathway has been reported to be the main signaling pathway that contributes to EGFR-mediated NB cell proliferation [19, 39, 40]. Therefore, we hypothesized that afatinib-induced cytotoxicity may result from the inhibition of EGFR-mediated PI3K/AKT/mTOR signaling. To test this hypothesis, we performed an EGF stimulation assay. As expected, afatinib blocked EGF-induced activation of EGFR/PI3K/AKT/mTOR signaling in all NB cell lines tested. These results show that EGFR inhibition by afatinib plays the major role in afatinib-induced cytotoxicity in NB cells. However, since non-EGFR ErbB family members were reported to contribute directly to NB growth and survival [20], inhibition of other ErbB family members by afatinib may have an auxiliary role in afatinib-induced cytotoxicity in NB. Afatinib is also known to target HER-2, but HER-2 expression is low or absent in NB and is known to not be necessary for NB tumorigenesis [20].

Development of chemoresistance is thought to be one of the main causes for relapse in cancer therapy and contributes largely to the poor outcome of high-risk NB patients. Therefore, understanding the molecular mechanisms for chemoresistance and developing new strategies to overcome chemoresistance are of vital importance in cancer treatment. Importantly, afatinib exhibits synergistic cytotoxicity with other compounds in a variety of cancer cells [41–43]. Therefore, we reasoned that afatinib may sensitize NB cells to traditional chemotherapy. In this study, we found that afatinib significantly enhanced doxorubicin-induced cytotoxicity in all the NB cell lines tested. Moreover, afatinib overcomes chemoresistance in the established chemoresistant LA-N-6 cell line, sensitizing LA-N-6 cells to doxorubicin treatment. These findings indicate that the combination of afatinib with the traditional therapeutic agent doxorubicin is superior to doxorubicin treatment alone and that the combination therapy of afatinib and doxorubicin is a potential effective strategy for NB therapy.

Tyrosine kinase inhibitors (TKIs) are a class of antagonists that target specific oncogenic tyrosine kinases and targeted therapy that aims to cure selected malignancies with the use of TKIs is an active field in cancer research [44]. The development of TKIs for ErbB family tyrosine kinase receptors has led to the use of several FDA-approved anti-cancer drugs such as gefitinib (Iressa, ZD1839), erlotinib (Tarceva, CP-358774, OSI-774), lapatinib (GW-572016), trastuzumab (Herceptin), and cetuximab (Erbitux) [45–48]. Gefitinib and erlotinib are selective EGFR tyrosine kinase inhibitors, and they are commonly used as the standard of care to treat patients with advanced NSCLC that harbor *EGFR* activating mutations. However, both of these inhibitors do not work in patients with the *EGFR* T790M activating mutation [49, 50]. Compared to wild type *EGFR*, proteins coded for with the gene with the T790M mutation have a 5-fold increased efficacy of kinase activation [51]; this increased activity is responsible for the poor outcome of lung cancer patients [52]. Lapatinib is a specific and reversible TKI of both EGFR and HER2 and shows off-target effects beyond these targets [53]. Despite the encouraging improvements in clinical outcomes with these EGFR inhibitors, side effects like acneiform rash were commonly observed [54]. In contrast to the first-generation reversible EGFR TKIs, the irreversible, pan-ErbB family inhibitor afatinib overcomes *EGFR* T790M mutation-driven resistance in NSCLC patients [55]. Afatinib is 100-fold more active against gefitinib-resistant tumors harboring the L858R-T790M *EGFR* mutation by covalently interacting with the mutant protein and irreversibly inhibiting its enzymatic activity [23]. In this study, we found that afatinib exhibited anti-tumor efficacy *in vitro* and *in vivo* by inducing apoptosis and blocking EGFR-mediated PI3K/AKT/mTOR signaling. Our study suggests that EGFR is a potential therapeutic target in NB and that afatinib could be used in the clinic to treat this devastating disease.

In summary, by using a panel of NB cell lines and an orthotopic mouse model of NB, we provide compelling evidence that afatinib is able to inhibit proliferation and promote apoptosis of NB cells. Although the role of non-EGFR ErbB signaling in afatinib-induced toxicity in NB needs to be further clarified, our findings broaden the therapeutic index of afatinib and provide preclinical evidence for the use of afatinib alone or in combination with the traditional therapeutic agent doxorubicin for NB patients.

## MATERIALS AND METHODS

### Antibodies and reagents

Small molecule inhibitor afatinib was purchased from LC Labs (A-8644) (LC Laboratories, Woburn, MA, USA). Recombinant human EGF was purchased from R&D systems (236-EG) (R&D Systems Inc., Minneapolis, MN, USA) and was prepared according to the manufacturer's recommendation. Doxorubicin (doxorubicin, D1515) and anti-β-Actin (A2228) antibodies were purchased from Sigma (Sigma-Aldrich Corp, St. Louis, MO, USA). Anti-phospho-EGFR (Y1068) (3777S), anti-EGFR (2232S), anti-phosphor-AKT (S473) (4060S), anti-AKT (9272S), anti-phospho-S6 (S235/236) (4858S), anti-S6 (2217S), anti-PARP (9532S), and anti-Caspase 3 (9662) primary antibodies, together with anti-Mouse (7076S) and anti-Rabbit (7074S) secondary antibodies were from Cell Signaling Technology (Cell Signaling Technology, Danvers, MA, USA).

### Cell lines and cell culture

Five of the six NB cell lines (IMR-32, NGP, NB-19, SK-N-AS, and SH-SY5Y) were cultured in RPMI Medium 1640 (RPMI) (Lonza, Walkersville, MD, USA) supplemented with 10% (v/v) heat-inactivated Fetal Bovine Serum (FBS) (SAFC Biosciences, Lenexa, KS, USA), 100 units/mL penicillin, and 100 μg/mL streptomycin. The chemoresistant NB cell line LA-N-6 was grown in RPMI containing 20% (v/v) heat-inactivated FBS, 100 units/mL penicillin, and 100 μg/mL streptomycin. All cells were cultured at 37°C in a humidified incubator with 5% CO_2_. All experiments were performed with cells under exponential growth conditions. The SH-SY5Y cell line with stable expression of luciferase was generated by transfection with a pcDNA3 luciferase expression plasmid into the cells. After 10 days of 800 μg/ml G418 (Enzo Life Sciences, Farmingdale, NY, USA) selection, a SH-SY5Y-luciferase stable cell line was obtained and used in the establishment of the orthotopic xenograft NB mouse model.

### Cell viability assay

Cell viability assay was performed as previously described [56, 57]. The Cell Counting Kit-8 (CCK-8) (Dojindo Laboratories, Rockville, MA, USA) was used according to the manufacturer's instructions. Cells were seeded in 96-well plates at density of 1 × 10^4^ cells per well. After 24 hrs of incubation at 37°C, the media were changed and the cells were treated with various concentrations of afatinib, doxorubicin, or their combinations for 48 hrs or 72 hrs. At the end of the treatment, cells were photographed and a mixture of 10 μL of CCK-8 and 190 μL of RPMI with 10% FBS was added into each well. Two hours later, the absorbance was measured at 450 nm using a microplate reader. Each experiment was performed in six replicates and the background reading of the media was subtracted from each well to standardize the results.

### Anchorage-independent growth assay

Cell anchorage-independent growth ability was assessed by soft agar assay performed as described previously [58, 59]. Briefly, a mixture of the autoclaved 5% base agar (in 56°C water bath) with RPMI containing 10% FBS was used to make the 0.5% (w/v) bottom agar. And 2 ml prepared bottom agar was added to each well till cooled down to semi-solid. For the top layer, 1.5 ml 0.3% agar was added to each well by mixing base agar with RPMI containing 10% FBS, together with the counted NB cells at the density of 1 × 10^4^ cells per well. Cells in culture were treated with the indicated concentrations of afatinib the next day and were maintained at 37°C for 2 to 3 weeks before staining with 500 μL of 0.005% crystal violet (C3886, Sigma) for 4 hrs. Images were captured by the microscope, and colonies were counted by Quantity One software (Bio-Rad Laboratories, Inc., Hercules, CA, USA) 4 hrs later. Each assay was performed in triplicate.

### Immunoblotting

The experiments were performed as described previously [60, 61]. Briefly, cells after treatment were washed with ice cold PBS twice and lysed at 4°C for 30 min in cooled RIPA buffer (50 mM Tris-HCl at pH 7.4, 150 mM NaCl, 1 mM EDTA, 1% NP-40, 0.25% sodium deoxycholate, 1 mM phenylmethylsulfonyl fluoride, 1 mM benzamidine, 10 μg/mL leupeptin, 1 mM dithiothreitol, 50 mM sodium fluoride, 0.1 mM sodium orthovanadate, and phosphatase inhibitor cocktail 2 and 3 (p5726 and p0044, Sigma)) on a rotator at 4°C for 30 min. The lysates were centrifuged at 13,000 rpm for 15 min and the supernatants were collected. Protein concentrations were measured using Bradford reagent (Bio-Rad Laboratories, Hercules, CA, USA). The supernatants were subjected to SDS-PAGE and then transferred to polyvinylidence fluoride (PVDF) membranes (BioRad), blocked with 5% milk or BSA at room temperature for one hour, and probed with appropriate dilutions of indicated primary antibodies overnight at 4°C. The membranes were then incubated with anti-mouse or rabbit IgG conjugated with horseradish peroxidase at room temperature for 1h. The membranes were developed using the ECL-Plus Western detection system (GE Health Care, Buckinghamshire, UK) according to the manufacturer's instruction. β-Actin was used as a loading control for whole cell extracts.

### EGF stimulation of NB cells

Five of the six NB cell lines (IMR-32, NGP, NB-19, SK-N-AS, and SH-SY5Y) were plated and grown in RPMI-1640 medium supplemented with 10% FBS (v/v) for 24 hrs. The chemoresistant LA-N-6 cell line were kept in RPMI-1640 medium supplemented with 20% FBS (v/v). The medium was then changed to FBS-free RPMI-1640 medium for 16 hrs. The six serum starved NB cells were treated with afatinib (10 μM) for one hour before exposed to serum-free RPMI-1640 medium with 100 ng/ml hEGF for 10 min. At the end of treatment, cells were collected and protein immunoblotting was performed as indicated.

### Antitumor efficacy of afatinib in an orthotopic xenograft NB mouse model

Five to six-week-old female athymic NCR nude mice were purchased from Taconic (Taconic, Hudson, NY, USA) and maintained under barrier conditions (pathogen-free conditions provided by plastic cages with sealed air filters). The preclinical xenograft mouse model of NB was established using an orthotopic (intrarenal) implantation of the NB cells as described previously [62–64]. Briefly, 1.5 × 10^6^ human luciferase-transduced SH-SY5Y cells was kept in 0.1 mL of PBS and a transverse incision was generated over the left flank of the nude mouse. And then the SH-SY5Y-luciferase cells were surgically injected into the left renal capsule and toward the superior pole of the left kidney of the nude mice. After engrafting for four weeks, mice with similar tumor sizes (using bioluminescent imaging to monitor tumor growth) were randomly divided into two groups: afatinib treated group (25 mg/kg by i.p. injection once daily for 3 days) and DMSO control group.

Four hours after the last injection the mice were sacrificed and the tumors were harvested and lysed for protein immunoblotting. All mice were handled according to protocols approved by the Institutional Animal Care and Use Committee of the Baylor College of Medicine.

### Statistical analysis

All values were presented as mean ± standard deviation (SD). Student's t-test was used to determine the statistical significance in all assays. A *P* <0.05 (*) was considered to be statistically significant. Each assay was repeated for at least three times and the representative results were presented.

## References

[1] Brodeur GM (2003). Neuroblastoma: biological insights into a clinical enigma. Nature reviews Cancer.

[2] Irwin MS, Park JR (2015). Neuroblastoma: paradigm for precision medicine. Pediatr Clin North Am.

[3] Louis CU, Shohet JM (2015). Neuroblastoma: molecular pathogenesis and therapy. Annu Rev Med.

[4] Nicolai S, Pieraccioli M, Peschiaroli A, Melino G, Raschella G (2015). Neuroblastoma: oncogenic mechanisms and therapeutic exploitation of necroptosis. Cell Death Dis.

[5] Smith MA, Seibel NL, Altekruse SF, Ries LA, Melbert DL, O'Leary M, Smith FO, Reaman GH (2010). Outcomes for children and adolescents with cancer: challenges for the twenty-first century. J Clin Oncol.

[6] Maris JM, Hogarty MD, Bagatell R, Cohn SL (2007). Neuroblastoma. Lancet.

[7] Hynes NE, Lane HA (2005). ERBB receptors and cancer: the complexity of targeted inhibitors. Nat Rev Cancer.

[8] Sequist LV, Soria JC, Goldman JW, Wakelee HA, Gadgeel SM, Varga A, Papadimitrakopoulou V, Solomon BJ, Oxnard GR, Dziadziuszko R, Aisner DL, Doebele RC, Galasso C (2015). Rociletinib in EGFR-mutated non-small-cell lung cancer. N Engl J Med.

[9] Brands RC, Muller-Richter UD, De Donno F, Seher A, Mutzbauer G, Linz C, Kubler AC, Hartmann S (2016). Co-treatment of wild-type EGFR head and neck cancer cell lines with afatinib and cisplatin. Mol Med Rep.

[10] Bellizzi A, Greco MR, Rubino R, Paradiso A, Forciniti S, Zeeberg K, Cardone RA, Reshkin SJ (2015). The scaffolding protein NHERF1 sensitizes EGFR-dependent tumor growth, motility and invadopodia function to gefitinib treatment in breast cancer cells. Int J Oncol.

[11] Lewandowska MA, Czubak K, Klonowska K, Jozwicki W, Kowalewski J, Kozlowski P (2015). The use of a two-tiered testing strategy for the simultaneous detection of small EGFR mutations and EGFR amplification in lung cancer. PLoS One.

[12] Lynch TJ, Bell DW, Sordella R, Gurubhagavatula S, Okimoto RA, Brannigan BW, Harris PL, Haserlat SM, Supko JG, Haluska FG, Louis DN, Christiani DC, Settleman J (2004). Activating mutations in the epidermal growth factor receptor underlying responsiveness of non-small-cell lung cancer to gefitinib. N Engl J Med.

[13] Walker F, Abramowitz L, Benabderrahmane D, Duval X, Descatoire V, Henin D, Lehy T, Aparicio T (2009). Growth factor receptor expression in anal squamous lesions: modifications associated with oncogenic human papillomavirus and human immunodeficiency virus. Hum Pathol.

[14] Kuan CT, Wikstrand CJ, Bigner DD (2001). EGF mutant receptor vIII as a molecular target in cancer therapy. Endocr Relat Cancer.

[15] Harris RC, Chung E, Coffey RJ (2003). EGF receptor ligands. Exp Cell Res.

[16] Ojeda SR, Ma YJ, Rage F (1997). The transforming growth factor alpha gene family is involved in the neuroendocrine control of mammalian puberty. Mol Psychiatry.

[17] Seshacharyulu P, Ponnusamy MP, Haridas D, Jain M, Ganti AK, Batra SK (2012). Targeting the EGFR signaling pathway in cancer therapy. Expert opinion on therapeutic targets.

[18] Colomiere M, Ward AC, Riley C, Trenerry MK, Cameron-Smith D, Findlay J, Ackland L, Ahmed N (2009). Cross talk of signals between EGFR and IL-6R through JAK2/STAT3 mediate epithelial-mesenchymal transition in ovarian carcinomas. British journal of cancer.

[19] Ho R, Minturn JE, Hishiki T, Zhao H, Wang Q, Cnaan A, Maris J, Evans AE, Brodeur GM (2005). Proliferation of human neuroblastomas mediated by the epidermal growth factor receptor. Cancer Res.

[20] Richards KN, Zweidler-McKay PA, Van Roy N, Speleman F, Trevino J, Zage PE, Hughes DP (2010). Signaling of ERBB receptor tyrosine kinases promotes neuroblastoma growth *in vitro* and *in vivo*. Cancer.

[21] Tamura S, Hosoi H, Kuwahara Y, Kikuchi K, Otabe O, Izumi M, Tsuchiya K, Iehara T, Gotoh T, Sugimoto T (2007). Induction of apoptosis by an inhibitor of EGFR in neuroblastoma cells. Biochem Biophys Res Commun.

[22] Dungo RT, Keating GM (2013). Afatinib: first global approval. Drugs.

[23] Li D, Ambrogio L, Shimamura T, Kubo S, Takahashi M, Chirieac LR, Padera RF, Shapiro GI, Baum A, Himmelsbach F, Rettig WJ, Meyerson M, Solca F (2008). BIBW2992, an irreversible EGFR/HER2 inhibitor highly effective in preclinical lung cancer models. Oncogene.

[24] Solca F, Dahl G, Zoephel A, Bader G, Sanderson M, Klein C, Kraemer O, Himmelsbach F, Haaksma E, Adolf GR (2012). Target binding properties and cellular activity of afatinib (BIBW 2992), an irreversible ErbB family blocker. J Pharmacol Exp Ther.

[25] Modjtahedi H, Cho BC, Michel MC, Solca F (2014). A comprehensive review of the preclinical efficacy profile of the ErbB family blocker afatinib in cancer. Naunyn Schmiedebergs Arch Pharmacol.

[26] Harbeck N, Solca F, Gauler TC (2014). Preclinical and clinical development of afatinib: a focus on breast cancer and squamous cell carcinoma of the head and neck. Future Oncol.

[27] De Pauw I, Wouters A, Van den Bossche J, Peeters M, Pauwels P, Deschoolmeester V, Vermorken JB, Lardon F (2016). Preclinical and clinical studies on afatinib in monotherapy and in combination regimens: Potential impact in colorectal cancer. Pharmacol Ther.

[28] Cappuzzo F, Finocchiaro G, Grossi F, Bidoli P, Favaretto A, Marchetti A, Valente ML, Cseh A, Clementi L, Massey D, Santoro A (2015). Phase II study of afatinib, an irreversible ErbB family blocker, in EGFR FISH-positive non-small-cell lung cancer. J Thorac Oncol.

[29] Zhan WJ, Zhu JF, Wang LM (2016). Inhibition of proliferation and induction of apoptosis in RB116 retinoblastoma cells by afatinib treatment. Tumour Biol.

[30] Tang Y, Zhang X, Qi F, Chen M, Li Y, Liu L, He W, Li Z, Zu X (2015). Afatinib inhibits proliferation and invasion and promotes apoptosis of the T24 bladder cancer cell line. Exp Ther Med.

[31] Chao TT, Wang CY, Chen YL, Lai CC, Chang FY, Tsai YT, Chao CH, Shiau CW, Huang YC, Yu CJ, Chen KF (2015). Afatinib induces apoptosis in NSCLC without EGFR mutation through Elk-1-mediated suppression of CIP2A. Oncotarget.

[32] Daneshmanesh AH, Hojjat-Farsangi M, Moshfegh A, Khan AS, Mikaelsson E, Osterborg A, Mellstedt H (2015). The PI3K/AKT/mTOR pathway is involved in direct apoptosis of CLL cells induced by ROR1 monoclonal antibodies. Br J Haematol.

[33] Meyers MB, Shen WP, Spengler BA, Ciccarone V, O'Brien JP, Donner DB, Furth ME, Biedler JL (1988). Increased epidermal growth factor receptor in multidrug-resistant human neuroblastoma cells. J Cell Biochem.

[34] Mendelsohn J, Baselga J (2006). Epidermal growth factor receptor targeting in cancer. Semin Oncol.

[35] Izycka-Swieszewska E, Brzeskwiniewicz M, Wozniak A, Drozynska E, Grajkowska W, Perek D, Balcerska A, Klepacka T, Limon J (2010). EGFR, PIK3CA and PTEN gene status and their protein product expression in neuroblastic tumours. Folia Neuropathol.

[36] Pugh TJ, Morozova O, Attiyeh EF, Asgharzadeh S, Wei JS, Auclair D, Carter SL, Cibulskis K, Hanna M, Kiezun A, Kim J, Lawrence MS, Lichenstein L (2013). The genetic landscape of high-risk neuroblastoma. Nat Genet.

[37] Eleveld TF, Oldridge DA, Bernard V, Koster J, Daage LC, Diskin SJ, Schild L, Bentahar NB, Bellini A, Chicard M, Lapouble E, Combaret V, Legoix-Ne P (2015). Relapsed neuroblastomas show frequent RAS-MAPK pathway mutations. Nat Genet.

[38] Keller J, Nimnual AS, Varghese MS, VanHeyst KA, Hayman MJ, Chan EL (2016). A Novel EGFR Extracellular Domain Mutant, EGFRDelta768, Possesses Distinct Biological and Biochemical Properties in Neuroblastoma. Mol Cancer Res.

[39] Evangelopoulos ME, Weis J, Kruttgen A (2005). Signalling pathways leading to neuroblastoma differentiation after serum withdrawal: HDL blocks neuroblastoma differentiation by inhibition of EGFR. Oncogene.

[40] Evangelopoulos ME, Weis J, Kruttgen A (2009). Mevastatin-induced neurite outgrowth of neuroblastoma cells via activation of EGFR. J Neurosci Res.

[41] Watanuki Z, Kosai H, Osanai N, Ogama N, Mochizuki M, Tamai K, Yamaguchi K, Satoh K, Fukuhara T, Maemondo M, Ichinose M, Nukiwa T, Tanaka N (2014). Synergistic cytotoxicity of afatinib and cetuximab against EGFR T790M involves Rab11-dependent EGFR recycling. Biochem Biophys Res Commun.

[42] Ioannou N, Seddon AM, Dalgleish A, Mackintosh D, Modjtahedi H (2013). Treatment with a combination of the ErbB (HER) family blocker afatinib and the IGF-IR inhibitor, NVP-AEW541 induces synergistic growth inhibition of human pancreatic cancer cells. BMC Cancer.

[43] Chen G, Noor A, Kronenberger P, Teugels E, Umelo IA, De Greve J (2013). Synergistic effect of afatinib with su11274 in non-small cell lung cancer cells resistant to gefitinib or erlotinib. PLoS One.

[44] Arora A, Scholar EM (2005). Role of tyrosine kinase inhibitors in cancer therapy. J Pharmacol Exp Ther.

[45] Shawver LK, Slamon D, Ullrich A (2002). Smart drugs: tyrosine kinase inhibitors in cancer therapy. Cancer Cell.

[46] Madhusudan S, Ganesan TS (2004). Tyrosine kinase inhibitors in cancer therapy. Clin Biochem.

[47] Roskoski R (2014). ErbB/HER protein-tyrosine kinases: Structures and small molecule inhibitors. Pharmacol Res.

[48] Cheng W, Hu Y, Sheng R (2014). Development of EGFR family small molecule inhibitors for anticancer intervention: an overview of approved drugs and clinical candidates. Curr Med Chem.

[49] Kuang Y, Rogers A, Yeap BY, Wang L, Makrigiorgos M, Vetrand K, Thiede S, Distel RJ, Janne PA (2009). Noninvasive detection of EGFR T790M in gefitinib or erlotinib resistant non-small cell lung cancer. Clin Cancer Res.

[50] Nguyen KS, Kobayashi S, Costa DB (2009). Acquired resistance to epidermal growth factor receptor tyrosine kinase inhibitors in non-small-cell lung cancers dependent on the epidermal growth factor receptor pathway. Clin Lung Cancer.

[51] Yun C-H, Mengwasser KE, Toms AV, Woo MS, Greulich H, Wong K-K, Meyerson M, Eck MJ (2008). The T790M mutation in EGFR kinase causes drug resistance by increasing the affinity for ATP. Proceedings of the National Academy of Sciences.

[52] Oxnard GR, Arcila ME, Chmielecki J, Ladanyi M, Miller VA, Pao W (2011). New strategies in overcoming acquired resistance to epidermal growth factor receptor tyrosine kinase inhibitors in lung cancer. Clinical cancer research.

[53] Dolloff NG, Mayes PA, Hart LS, Dicker DT, Humphreys R, El-Deiry WS (2011). Off-target lapatinib activity sensitizes colon cancer cells through TRAIL death receptor up-regulation. Sci Transl Med.

[54] Dancey JE, Freidlin B (2003). Targeting epidermal growth factor receptor--are we missing the mark?. Lancet.

[55] Bordi P, Tiseo M, Bortesi B, Naldi N, Buti S, Ardizzoni A (2014). Overcoming T790M-driven acquired resistance to EGFR-TKIs in NSCLC with afatinib: a case report. Tumori.

[56] Zhang H, Dou J, Yu Y, Zhao Y, Fan Y, Cheng J, Xu X, Liu W, Guan S, Chen Z, shi Y, Patel R, Vasudevan SA (2015). mTOR ATP-competitive inhibitor INK128 inhibits neuroblastoma growth via blocking mTORC signaling. Apoptosis.

[57] Li H, Chen Z, Hu T, Wang L, Yu Y, Zhao Y, Sun W, Guan S, Pang JC, Woodfield SE, Liu Q, Yang J (2016). Novel proteasome inhibitor ixazomib sensitizes neuroblastoma cells to doxorubicin treatment. Scientific Reports.

[58] Wang Y, Wang L, Guan S, Cao W, Wang H, Chen Z, Zhao Y, Yu Y, Zhang H, Pang JC, Huang SL, Akiyama Y, Yang Y (2016). Novel ALK inhibitor AZD3463 inhibits neuroblastoma growth by overcoming crizotinib resistance and inducing apoptosis. Sci Rep.

[59] Li H, Wang Y, Chen Z, Lu J, Pan J, Yu Y, Zhao Y, Zhang H, Hu T, Liu Q, Yang J (2016). Novel multiple tyrosine kinase inhibitor ponatinib inhibits bFGF-activated signaling in neuroblastoma cells and suppresses neuroblastoma growth *in vivo*. Oncotarget.

[60] Fan Y, Mao R, Yu Y, Liu S, Shi Z, Cheng J, Zhang H, An L, Zhao Y, Xu X, Chen Z, Kogiso M, Zhang D (2014). USP21 negatively regulates antiviral response by acting as a RIG-I deubiquitinase. J Exp Med.

[61] Liu ZG, Tang J, Chen Z, Zhang H, Wang H, Yang J, Zhang H (2016). The novel mTORC1/2 dual inhibitor INK128 enhances radiosensitivity of breast cancer cell line MCF-7. Int J Oncol.

[62] Chen Z, Wang Z, Pang JC, Yu Y, Bieerkehazhi S, Lu J, Hu T, Zhao Y, Xu X, Zhang H, Yi JS, Liu S, Yang J (2016). Multiple CDK inhibitor dinaciclib suppresses neuroblastoma growth via inhibiting CDK2 and CDK9 activity. Sci Rep.

[63] Shi Y, Ma IT, Patel RH, Shang X, Chen Z, Zhao Y, Cheng J, Fan Y, Rojas Y, Barbieri E, Chen Z, Yu Y, Jin J (2015). NSC-87877 inhibits DUSP26 function in neuroblastoma resulting in p53-mediated apoptosis. Cell Death Dis.

[64] Patterson DM, Shohet JM, Kim ES (2011). Preclinical models of pediatric solid tumors (neuroblastoma) and their use in drug discovery. Curr Protoc Pharmacol.

